# Abiotic and Landscape Factors Constrain Restoration Outcomes Across Spatial Scales of a Widespread Invasive Plant

**DOI:** 10.3389/fpls.2019.00481

**Published:** 2019-04-18

**Authors:** Christine B. Rohal, Chad Cranney, Karin M. Kettenring

**Affiliations:** Department of Watershed Sciences and the Ecology Center, Utah State University, Logan, UT, United States

**Keywords:** spatial scale, *Phragmites australis*, management, invasive species control, restoration, contingency

## Abstract

The natural recolonization of native plant communities following invasive species management is notoriously challenging to predict, since outcomes can be contingent on a variety of factors including management decisions, abiotic factors, and landscape setting. The spatial scale at which the treatment is applied can also impact management outcomes, potentially influencing plant assembly processes and treatment success. Understanding the relative importance of each of these factors for plant community assembly can help managers prioritize patches where specific treatments are likely to be most successful. Here, using effects size analyses, we evaluate plant community responses following four invasive *Phragmites australis* management treatments (1: fall glyphosate herbicide spray, 2: summer glyphosate herbicide spray, 3: summer imazapyr herbicide spray, 4: untreated control) applied at two patch scales (12,000 m^2^ and 1,000 m^2^) and monitored for 5 years. Using variation partitioning, we then evaluated the independent and shared influence of patch scale, treatment type, abiotic factors, and landscape factors on plant community outcomes following herbicide treatments. We found that *Phragmites* reinvaded more quickly in large patches, particularly following summer herbicide treatments, while native plant cover and richness increased at a greater magnitude in small patches than large. Patch scale, in combination with abiotic and landscape factors, was the most important driver for most plant responses. Compared with the small plots, large patches commonly had deeper and more prolonged flooding, and were in areas with greater hydrologic disturbance in the landscape, factors associated with reduced native plant recruitment and greater *Phragmites* cover. Small patches were associated with less flooding and landscape disturbance, and more native plants in the surrounding landscape than large patches, factors which promoted higher native plant conservation values and greater native plant cover and richness. Herbicide type and timing accounted for very little of the variation in native plant recovery, emphasizing the greater importance of patch selection for better management outcomes. To maximize the success of treatment programs, practitioners should first manage *Phragmites* patches adjacent to native plant species and in areas with minimal hydrologic disturbance.

## Introduction

Invasive plants can reduce the abundance and diversity of native plant communities, and can markedly alter ecosystem functions and services, making invasive plant management a restoration priority (Hejda et al., 2009). It is often assumed that desirable species will naturally recover once the invader is removed (Galatowitsch et al., 1999), but this is rarely the case (Kettenring and Adams, 2011). Restoration outcomes are notoriously variable across sites, and can be very challenging to predict (Suding, 2011; Brudvig et al., 2017). But clearly understanding the constraints to restoration success is important in order to prioritize sites for invasive species management that are most likely to have successful outcomes (i.e., effective removal of the invader and native plant recovery), or to plan for the additional efforts needed to overcome thresholds at sites that have a high degree of impairment (Suding, 2011).

The results of invasive species removal can be limited by a wide array of factors acting across spatial scales. Managers often focus on optimizing management regimes which can influence both invader removal success and plant community outcomes (Mason and French, 2007). Local abiotic factors can also influence the effectiveness of management tools, the likelihood for native plant recovery, and the competitive dynamics between native and invasive species (Diez et al., 2009). Landscape context, particularly the degree of landscape disturbance and the composition of the surrounding vegetation, can influence plant assembly trajectories following management (Prach and Hobbs, 2008; Reinecke et al., 2008; Matthews et al., 2017). To add a further layer, the spatial scale of a managed patch can have implications for restoration outcomes (Holl and Crone, 2004; Morrison et al., 2010), but this has been relatively unexplored (Brudvig, 2011).

It is often recognized that treatments enacted at small scales can be more effective at invader removal (Quirion et al., 2018) and result in more robust native plant recovery (Erskine Ogden and Rejmánek, 2005) than large-scale efforts. Larger scale treatments may have to be conducted with different methods than smaller patches (e.g., resource intensive methods such as manual removal of invasive plants are typically infeasible at large scales) which can have implications for success (Kettenring and Adams, 2011). The patch scale can also influence plant assembly processes following management treatments. The spatial heterogeneity of biotic and abiotic conditions within a patch is likely to increase with increasing patch area, leading to greater opportunities for a diversity of species recruitment in larger patches (De Blois et al., 2002; Englund and Cooper, 2003). A patch’s size might also influence its degree of openness (edge-to-area ratio), which can impact its permeability to the exchange of organisms (Englund and Cooper, 2003). Small patches that have a higher edge to interior ratio might be more influenced by propagules or clonal growth from the surrounding matrix and edge-mediated environmental conditions (e.g., light intensity) that influence plant community assembly patterns (Phillips and Shure, 1990; De Blois et al., 2002). The unique impact of patch size on community assembly is challenging to distinguish from the local and landscape factors that can co-occur with patch scale (De Blois et al., 2002; Pauchard and Shea, 2006). The abiotic environment of a patch can differ with its scale, in turn influencing species assembly (Phillips and Shure, 1990). In addition, landscape-scale disturbance factors that can enable the formation of large invaded patches (Zedler and Kercher, 2004) might then constrain the assembling plant community following management treatments (Ehrenfeld, 2008; Tousignant et al., 2010).

*Phragmites australis* (Cav.) Trin ex Steud (i.e., common reed, hereafter called *Phragmites*) is a widespread invasive plant present in wetlands across North America (Saltonstall, 2003; Kettenring et al., 2012) that is often the target of restoration efforts (Martin and Blossey, 2013; Rohal et al., 2018). The impacts of *Phragmites* to plant biodiversity (Chambers et al., 1999), wildlife habitat quality (Dibble et al., 2013), and ecosystem functioning (Findlay et al., 2003; Rooth et al., 2003) have led managers to spend large sums of money and time on its management (Martin and Blossey, 2013; Rohal et al., 2018). With the capacity to reproduce both sexually and asexually (Kettenring and Mock, 2012), *Phragmites* invades wetlands by colonizing new areas from seed or rhizome fragments, which can then rapidly expand through clonal growth (Amsberry et al., 2000; Kettenring et al., 2016). A disturbance specialist (Minchinton and Bertness, 2003), *Phragmites* often becomes established following a (small-scale) disturbance in a wetland (Kettenring et al., 2015), and expands most rapidly following additional disturbance to the vegetation matrix or hydrologic drawdowns (Warren et al., 2001). These processes can create a pattern across the landscape of small patches surrounded by remnant native vegetation intermixed with large-scale stands where patches have expanded and merged to form large monocultures (Lathrop et al., 2003).

Given the widespread nature of the *Phragmites* invasion and limited resources for restoration, managers must often prioritize sites, often choosing between targeting the large stands where the impacts appear most severe or focusing on the initial patches, which can spread rapidly (Moody and Mack, 1988; Hazelton et al., 2014). *Phragmites* managers must also select patches across heterogeneous abiotic conditions, which can impact treatment success (Rohal, 2018). And they must select patches within a diverse landscape, with different matrix vegetation and varying levels of landscape-scale disturbances (Long et al., 2017b). These abiotic and landscape influences may have contributed to *Phragmites*’ presence and therefore might further restrict restoration outcomes (Hazelton et al., 2014; Long et al., 2017a). Furthermore, managers must select the appropriate management action, which most commonly involves herbicide, primarily due to its cost-effectiveness (Martin and Blossey, 2013; Rohal et al., 2018). Manual methods such as burning and mowing that do not kill roots and rhizomes are not effective without the addition of herbicide, and biological control is presently unavailable (Hazelton et al., 2014). Managers must choose the timing of herbicide application, commonly in summer or fall, and the type of herbicide: imazapyr, or glyphosate (Hazelton et al., 2014). Spray timing and the composition of herbicide can have different effectiveness regarding *Phragmites* dieback and non-target native plant impacts (Mozdzer et al., 2008). Knowing the degree to which *Phragmites* management choices, patch scale, local abiotic factors, and landscape factors might independently and jointly influence restoration outcomes can help managers focus on the most influential factors constraining success. While restoration research often focuses on management and site level abiotic factors, an understanding of how these factors interact with patch scale and landscape factors to influence restoration outcomes is relatively unexplored (Brudvig, 2011; Grman et al., 2013).

Here we investigate the plant community outcomes of three different *Phragmites* management regimes (summer glyphosate, summer imazapyr, and fall glyphosate) at two discrete spatial scales (small 1000 m^2^ patches representing initial invasions, and large 12,000 m^2^ patches representing large stands). These three herbicide timings were chosen based on manager suggestions of the most logistically feasible treatments (Rohal et al., 2018) and the restoration ecology literature that indicated that these treatments might be most successful (Ailstock et al., 2001; Mozdzer et al., 2008). We asked, does the scale at which a treatment is conducted influence (1) *Phragmites* cover, (2) native perennial plant cover, and (3) species richness of the returning plant community? And is this effect (or lack of effect) consistent over a typical 5-year management time frame? We also sought to understand the influence of patch scale and management choices in relation to other factors known to influence assembling plant communities, specifically their local abiotic and landscape contexts. Using variance partitioning, we asked: what is the relative influence of patch scale, management, local abiotic factors, and landscape factors on assembling plant communities following *Phragmites* management? Are the differences we see in plant community outcomes at different spatial scales attributable to the patch scale alone? Or are there abiotic and landscape factors that are associated with patch scale that influence different plant community responses?

## Materials and Methods

### Study Sites

**FIGURE 1:**
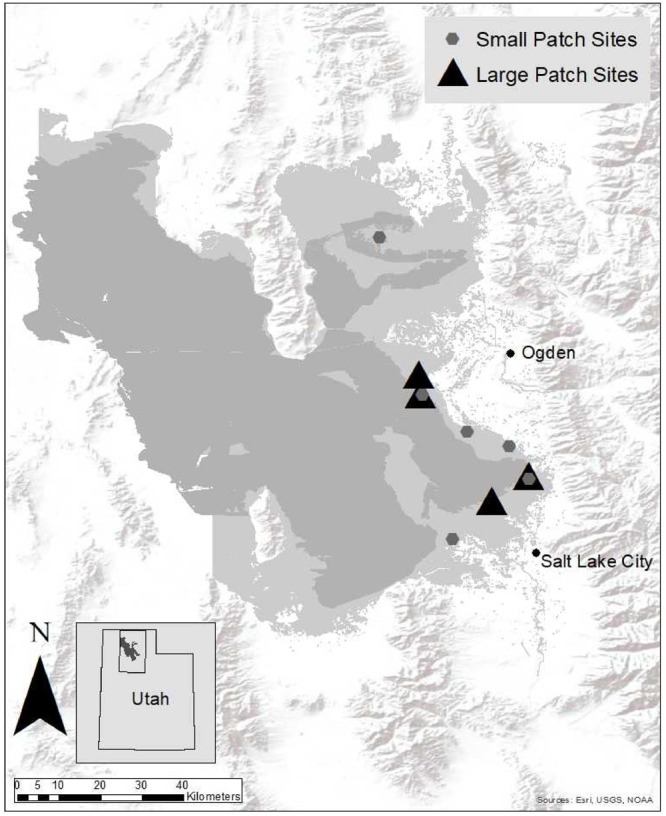
Sites in the wetlands on the eastern shore of Great Salt Lake, Utah, United States, where the small and large patch *Phragmites* management treatments took place. The dark gray shading indicates open water in GSL. The surrounding light gray shading outlines the adjacent wetland complexes.

This study was conducted in wetlands on the eastern shore of the Great Salt Lake (GSL), Utah (Figure 1). Invasive *Phragmites* became prominent in this region after a major flooding event in the mid-1980s (Rohal et al., 2018). Since then its footprint has expanded to over 93 km^2^ (Long et al., 2017a). *Phragmites* is present in very large, well-established stands isolated from native species, as well as small patches that are still surrounded by a matrix of native vegetation. Dominant vegetation in this region includes *Bolboschoenus maritimus, Schoenoplectus acutus, S. americanus, Distichlis spicata, Typha domingensis*, and *T. latifolia* (Downard et al., 2017).

### Treatments

We established 16 large-scale (12,000 m^2^) plots within four sites and 20 small-scale (1,000 m^2^) plots within six sites (Figure 1). Each plot was randomly assigned one treatment, such that all treatments were equally replicated (*n* = 9; *n* = 4 for treatments applied at large scales and *n* = 5 for treatments applied at small scales). Treatments were applied in only one plot per site (all treatments were not conducted at every small patch site due to space constraints and imazapyr permitting restrictions at some locations). Treatments were (1) summer imazapyr herbicide spray, (2) summer glyphosate herbicide spray, (3) fall glyphosate herbicide spray, and (4) untreated control. Due to space and logistical limitations, we were not able to include a fall imazapyr herbicide treatment. This treatment was less interesting to land managers that were consulted prior to experiment initiation (Rohal et al., 2018). All herbicide treatments were followed by a winter mow. Plots were established in areas that had ≥75% *Phragmites* cover which had not been managed in the previous 5 years. *Phragmites* cover was not significantly different in small and large patches (paired *T*-test: *P* = 0.39). Initial treatments were conducted in 2012, follow-up treatments conducted in 2013–2014, and monitoring continued in 2015–2016.

Summer herbicide treatments were conducted during the last week of June and first week of July. Fall herbicide treatments were conducted in the last week of August through first week of September. Initial herbicide treatments in 2012 were applied to both scales using a piston-driven sprayer on a boomless nozzle attached to Softrak wetland tractors (Loglogic, Mutterton, Cullompton, Devon, United Kingdom) or other marsh capable vehicles. At the large scales, follow-up treatments were applied using the same equipment, but from handheld nozzles which were used to treat individual patches to minimize non-target plant mortality. At the small scales, follow-up treatments were applied using backpack sprayers. We applied herbicides on sunny, non-windy days to avoid drift (average winds < 8 m/s), at the label-recommended rate of 7 L/hectare. We mixed herbicides with the non-ionic surfactant LI-700 at the label recommended rate of 1.89 L/378.54 L mixed solution. We mowed all herbicide treatment plots in the winters (when the marsh soil is frozen, allowing better access) of 2012 and 2013 to accelerate the decomposition of standing dead biomass. We conducted mowing using an ASV PT-80 skidsteer (ASV Inc., Grand Rapids, MN, United States) or a MarshMaster (Coast Machinery LLC, Baton Rouge, LA, United States) equipped with front-end hydraulic rotary mowers.

### Data Collection

At both scales, we monitored vegetation annually in September 2013–2016 after the fall herbicide treatments had been conducted. Pre-treatment monitoring occurred in June 2012 before the initial summer herbicide treatments were implemented. In the small patches, vegetation was sampled along four permanent, evenly spaced transects, within four, evenly spaced 1 m^2^ quadrats placed to the right of each transect. In the large patches, vegetation was sampled along two permanent, evenly spaced transects, with two 1 m^2^ quadrats placed on either side of each transect at 10 evenly-spaced locations. We determined percent cover by ocular estimation in each 1 m^2^ quadrat using cover classes (<1%, 1–5%, 6–25%, 26–50%, 51–75%, and 76–100%). Percent cover estimations were made by the same two individuals following a coordination test to ensure similar estimations. We identified plants to species level (Welsh et al., 1993) with up-to-date nomenclature identified using the USDA PLANTS database (USDA, NRCS, 2019).

We selected the 16 innermost quadrats from the large patch plots in order to calculate plant community metrics that were comparable to the small-scale plots. Species richness was calculated for both scales at the plot level. Then, a mean coefficient of conservatism (mean *C*) was calculated for each plot to estimate habitat quality. Coefficients of conservatism (CC) are expert-derived values that describe a plant species’ disturbance tolerance or habitat specificity (Cohen et al., 2004). Species are assigned conservation values from 0 to 10, where species with the highest CC exhibit the least tolerance to human disturbance, while zeros represent exotic or invasive species. Mean *C* is a robust metric frequently used to assess conservation value in wetlands (Matthews et al., 2005). We calculated mean *C* for all native species in each plot, using *C*-values developed for other Western states that were evaluated for suitability in Utah (Menuz et al., 2016).

### Local Abiotic and Landscape Predictor Variables

We collected soil samples in June 2012, before the initial treatments were conducted, to characterize local abiotic conditions in the small and large plots. In the small plots, we collected one soil sample at the midpoint of each transect (four samples per plot). In the large plots, we collected three samples at three evenly spaced intervals per transect (six samples per plot). We used a 7.62-diameter auger to collect a 30-cm deep sample of mineral soil after measuring and removing the organic horizon. The Utah State University soil analytics lab processed the soils for pH and electrical conductivity (Rhoades, 1982), and available phosphorus (Olsen NaHCO_3_ method). The USU Stable Isotope Lab assessed the soils’ total nitrogen by continuous-flow direct combustion and mass spectrometry (CF-IRMS). We recorded water depth measurements at every quadrat during vegetation sampling events. Water depth is known to be an important driving factor in wetland plant communities (Casanova and Brock, 2000), while other abiotic variables like nutrients and pH are known to influence the divergence in exotic and native species dominance (Ehrenfeld, 2008). Abiotic measurements were averaged by plot for analyses.

We derived most of the landscape predictor variables from publicly available spatial datasets in ArcGIS 10.2. We determined if each plot was positioned within an impounded wetland by consulting the National Wetlands Inventory classification for each plot location. Many GSL wetlands are impounded to mitigate water losses in wetlands due to upstream diversions for urban and agricultural uses, which stabilizes the availability of waterfowl habitat, but has implications for wetland condition (Downard et al., 2014). We used ArcGIS’s NEAR function which calculates distances between features to determine each plots distance to the nearest point source discharge using the locations of Utah Pollutant Discharge Elimination System (NPDES) permits from the Utah Department of Environmental Water Quality. Distance to UPDES permits was a significant factor associated with *Phragmites* presence in a GSL species distribution model (Long et al., 2017a).

We drew a 1000-m buffer (Matthews et al., 2009) around each plot from which we collected additional anthropogenic disturbance and land-use information. Landscape-level disturbances such as human development, roads, and agriculture have been correlated with plant invasions and other plant community changes in other wetlands (Chambers et al., 2008; Tousignant et al., 2010; Menuz and Kettenring, 2013). Within each buffer, we determined the length of roads and canals which were hand digitized using high resolution (0.5 m resolution) aerial imagery collected in June 2016. We overlaid each buffer with water diversion data, sourced from the Utah Division of Water Rights, to determine the number of water diversions in each 1000 m radius surrounding each plot. Water diversions, roads (often associated with dikes), and canals represented the degree of hydrologic manipulation in the surrounding region. We determined the proportion of agriculture, developed land, open water, and emergent wetlands within each 1000 m buffer using the National Land Cover Dataset.

Variables related to the vegetation matrix are correlates for dispersal of propagules entering the plot. Plots with high levels of *Phragmites* cover in the matrix should be expected to receive high levels of propagule pressure influencing further *Phragmites* invasion (Simberloff, 2009), while plots surrounded by high levels of native species should have more native propagules available to assemble (Palmer et al., 1997). Long distance dispersal (particularly for water-transported seed like many of Utah’s native wetland species) may be disrupted by other factors, such as man-made impoundments and water control structures (Soomers et al., 2013). Around the small plots, we collected data on the surrounding vegetation matrix of each plot in summer 2013. We expected the vegetation in these areas to remain relatively stable year-to-year in the absence of disturbance. We placed 12 1 m^2^ quadrats at even intervals 7 m outside of the plot edge, and collected cover data for each species using the same cover classes used within plots. Around the large plots, we used high resolution 4-band (RGB–red, blue, green, + NIR–near infrared) aerial imagery collected in the summer of 2013 to determine the vegetation composition at 12 even intervals 7 m from the edge of each plot. Our analysis determined that each large plot was surrounded by *Phragmites* monocultures, which was confirmed during site visits.

### Data Analysis

To evaluate if vegetation metrics varied between large and small patches, we conducted separate effect size analyses for each treatment, at each scale, within each monitoring year. The effect size approach enabled us to evaluate if the direction and magnitude of treatment effects varied between small and large patches (Rinella and James, 2010). For our effect size statistic, we calculated natural log response ratio (lnRR) of *Phragmites* cover and native perennial cover where lnRR for each site = ln(treated/control). We performed a meta-analysis of each variable using the metafor package in R (Viechtbauer, 2010). We analyzed effect sizes with the RMA function and restricted maximum-likelihood (REML) method. We used mixed-effects models, with patch as the random effect, to test the significance (α = 0.05) of effect size estimates (*z*-test; H0: *m* = 0) from each treatment, at each scale, within each year, and examine the difference between scales during each year (*Q*_M_-test; H0: *b*_1_ = *b*_2_ = 0) (Monaco et al., 2018).

To understand the relative influence of patch scale, management, abiotic factors, and landscape factors on assembling plant communities, we conducted principal components analyses (PCAs), variation partitioning, and redundancy analyses in R 3.0.2 (R Development Core Team, 2013) using package vegan 2.2-1 (Oksanen et al., 2015). For these analyses, we used data from all herbicide treated plots (27 total plots; 12 large scale, 15 small scale) from 2015, the year following the last herbicide treatments. We removed rare species and used a Hellinger’s transformation on our plant community data. Hellinger’s transformations on community datasets with large numbers of zeros enable the use of linear methods such as redundancy analysis (RDA) (Legendre and Gallagher, 2001). We transformed some univariate predictor variables and response variables to best meet the assumptions of normality. We log-transformed abiotic variables phosphorus, nitrogen, average water depth (across all monitoring years), and 2015 water depth (depth during the year selected for analysis). We log-transformed landscape variables including the length of canals, length of roads, and distance to the nearest discharge. We arcsine-square-root-transformed the proportion of development, proportion of agriculture, and cover of surrounding native perennials. We square-root-transformed the distance to nearest water diversion variable. We logit-transformed the response variables *Phragmites* cover and native perennials cover. We then reduced local abiotic and landscape disturbance variables in separate PCAs in order to reduce redundant variables and address collinearity (Graham, 2003). For the landscape category, we excluded the proportion of surrounding emergent marsh (1 km) and percent cover of native perennials in the surrounding area from the PCA. These variables were less related to landscape disturbance and were more reflective of surrounding marsh conditions. They were included as separate variables in further analysis.

After evaluating the resulting variables for collinearity (Supplementary Table S1), we performed stepwise regressions to reduce the number of predictor variables within each variable set to be used in each separate variation partitioning analysis (Grman et al., 2013). For plant community data, we used the function OrdiR2step to run significance tests on each separate variable set, retaining predictors with *P* < 0.1, and used adjusted *R*^2^ as a selection criteria to avoid overfitting (Blanchet et al., 2008). For univariate response variables, we used the MASS package (Venables and Ripley, 2002) to reduce variables in each variable set, using AIC to compare among models. If the stepwise procedure resulted in no selected variables, we manually selected the variable with the smallest increase in AIC, or adjusted *R*^2^, to ensure each variable set had at least one predictor variable to perform variation partitioning. To evaluate the significance of the variables included in the final models, we used permutation tests for marginal significance (RDA for plant community composition) and marginal sums of squares (multiple regression for univariate responses).

We then performed separate variation partitioning (Legendre and Legendre, 2012) analyses for each response variable using RDA with the function varpart. Factors across hierarchical scales are often intercorrelated, which can make it challenging to disentangle the independent effects of possible causal variables (Matthews et al., 2009). Variation partitioning among sets of explanatory variables is a method that evaluates the relative importance and confounding of factors across multiple hierarchical levels (Cushman and McGarigal, 2002). The variation partitioning procedure uses partial ordinations to determine (1) the amount of variation independently attributable to each set of predictors after accounting for the effects of the other sets of predictors and (2) the amount of variation shared among sets of predictors (Matthews et al., 2009). We used adjusted *R*^2^ as an unbiased estimate of variation explained which allows for the comparison of sets with different numbers of predictor variables (Peres-Neto et al., 2006). Testable fractions (the unique variable sets) were evaluated for significance using ANOVA in the package vegan. We repeated analyses without reducing variables using variable selection and saw the same patterns of variation explained across explanatory sets.

## Results

### Effects Size Analysis

**FIGURE 2:**
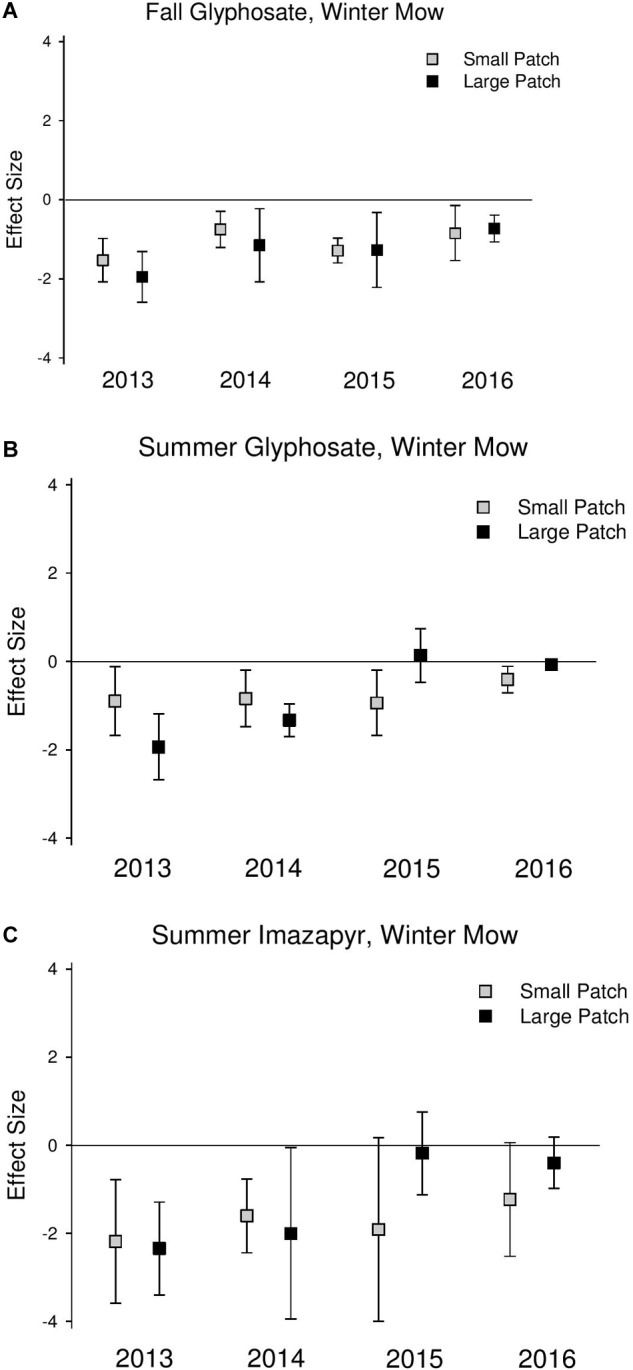
Effect size graphs for *Phragmites* cover for three herbicide treatments [**(A)** fall glyphosate, winter mow, **(B)** summer glyphosate, winter mow, and **(C)** summer imazapyr, winter mow]. Points are effect sizes at each scale, in each year, bounded by the upper and lower limits of the 95% confidence interval.

*Phragmites* cover was significantly reduced at similar magnitudes in the first 2 years following all initial treatments (2013 and 2014) across both scales (Figure 2 and Supplementary Table S2). In 2015 and 2016, 1 and 2 years after the final herbicide treatments, *Phragmites* cover was reduced at a greater magnitude in the small patches compared to the large patches across all treatments, but the difference was only significant in the summer glyphosate treatment (2015 *Q*_M_ = 4.85, *P* = 0.03; 2016 *Q*_M_ = 4.57, *P* = 0.03; Supplementary Table S2) due to wide confidence intervals, indicating large amounts of site variability.

**FIGURE 3:**
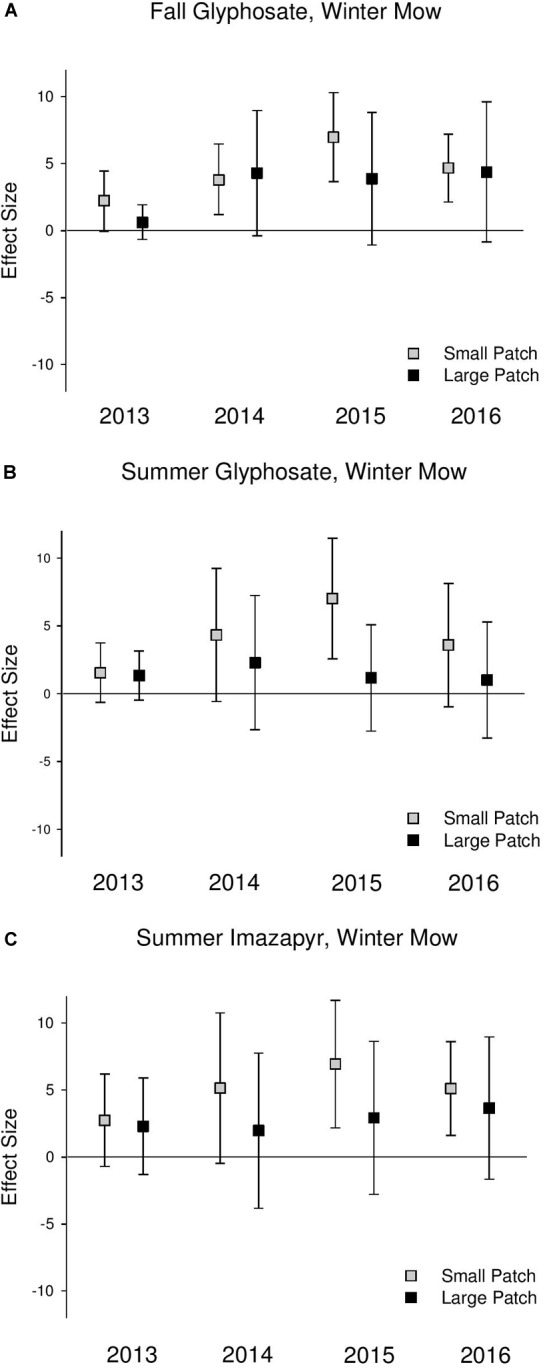
Effect size graphs for native emergent perennial cover for three herbicide treatments [**(A)** fall glyphosate, winter mow, **(B)** summer glyphosate, winter mow, and **(C)** summer imazapyr, winter mow]. Points are effect sizes at each scale, in each year, bounded by the upper and lower limits of the 95% confidence interval.

**FIGURE 4:**
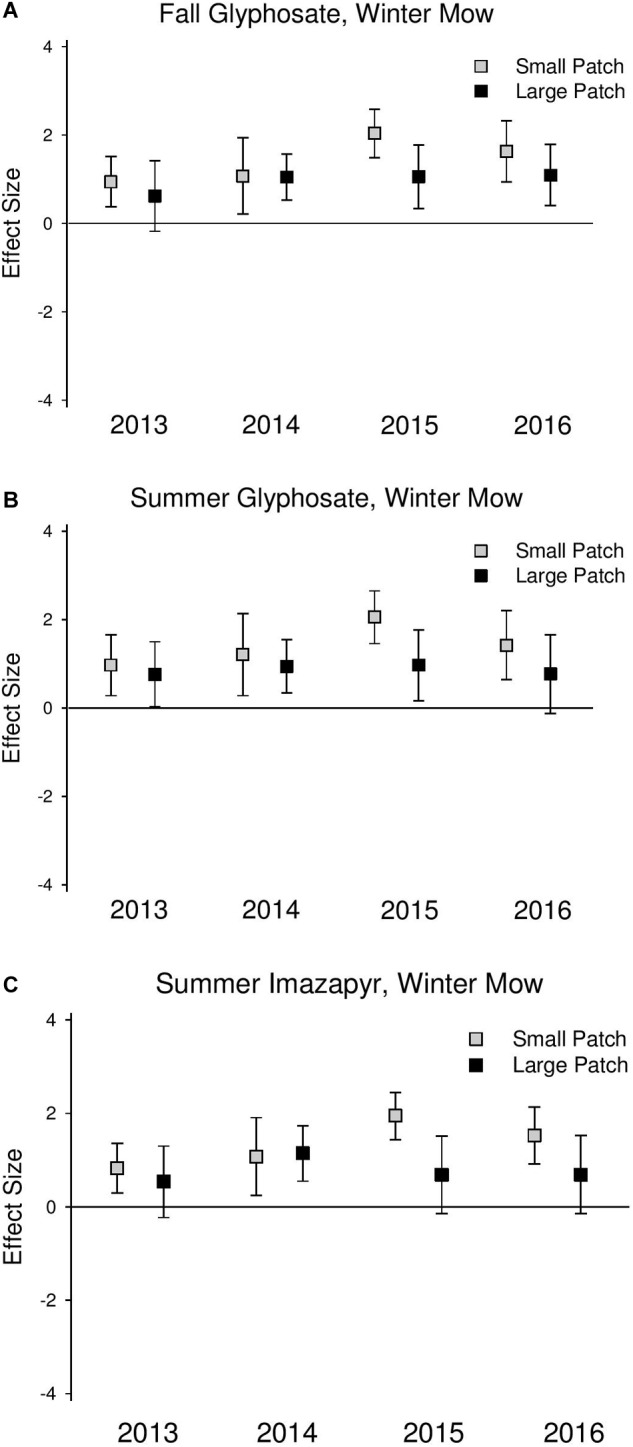
Effect size graphs for species richness for three herbicide treatments [**(A)** fall glyphosate, winter mow, **(B)** summer glyphosate, winter mow, and **(C)** summer imazapyr, winter mow]. Points are effect sizes at each scale, in each year, bounded by the upper and lower limits of the 95% confidence interval.

Native perennial cover increased across both scales following all treatments, but this effect was never significant at the large scales (confidence intervals always overlapped 0) (Figure 3 and Supplementary Table S3). Native perennial cover significantly increased in small-scale patches across all treatments in most years in 2014–2016, and the increase was consistently at a greater magnitude than large patches, though the difference was only significant in the summer glyphosate treatment in 2015 (2015 *Q*_M_ = 3.79, *P* = 0.05; Supplementary Table S3). Species richness significantly increased across both scales following treatments, but at a consistently higher magnitude in the small-scale patches than large (Figure 4 and Supplementary Table S4).

### Variable Reduction

The reduction of abiotic variables resulted in two retained principal components that explained 69.1% of the variation in abiotic factors among patches (Table 1). PC axis 1 loaded heavily on water depth variables and organic horizon depth, which generally is higher with deeper and prolonged flooding (Reddy and DeLaune, 2008). This axis primarily represented a hydrologic gradient from drier conditions to deeper water conditions. This axis also represented a gradient from low to high salinity. PC axis 2 loaded most heavily on phosphorus and nitrogen and primarily represented a nutrient gradient.

**Table 1:** Variable loadings on principal components axes.

Variable	PC axis 1	PC axis 2
**Abiotic variables**		
pH	–	–0.417
Salinity	0.482	–
Phosphorus	–	0.602
Nitrogen	–0.186	0.631
Water depth 2015	0.512	0.163
Average water depth 2012–2016	0.541	0.181
O horizon depth	0.423	–
Variance explained (%)	40.13	28.93
**Landscape disturbance variables**		
Impounded	0.377	0.290
Proportion developed land (1 km)	–0.211	0.586
Proportion agriculture (1 km)	–0.339	0.386
Length of canals (1 km)	0.475	0.296
Length of roads (1 km)	0.393	0.263
Distance to nearest discharge	0.477	0.109
Number of water diversions (1 km)	–0.300	0.505
Variance explained (%)	44.82	27.99

The reduction of landscape disturbance variables resulted in two retained principal components that explained 72.8% of the variation in landscape variables among patches (Table 1). PC axis 1 loaded most heavily on the canals and roads, impoundment, and distance to the nearest discharge. This axis primarily represented a gradient in the degree of hydrologic and water quality manipulation in the surrounding landscape. PC axis 2 loaded most heavily on water diversions, proportion of developed land, and proportion of agriculture, which suggests this axis primarily represents a gradient of human infrastructure in the surrounding landscape.

Stepwise variable selection retained abiotic PCA1 (describing plot hydrology) for three response variables; the factors in abiotic PCA1 had a significant negative effect on species richness, mean *C*, and percent cover of native perennials (Table 2). Abiotic PCA2 (nutrients) was not selected in variable selection for any response variable. Landscape PCA1 (describing landscape hydrologic disturbance) was retained in all models except mean *C*; the factors in PCA1 had a negative influence on species richness and native perennial cover and a positive influence on *Phragmites* cover. Percent cover surrounding natives had a significant positive effect on all response variables except *Phragmites* (Table 2). Landscape PCA2 was only significant for the plant community model. The proportion of emergent marsh in the surrounding area had a positive effect on species richness and a negative effect on *Phragmites* cover, though this effect was only moderately significant. Herbicide season was significant for both *Phragmites* and mean *C* models, while herbicide type was not significant in all models. Spatial scale was selected and significant in all models except for the plant community analysis. The direction of spatial effects indicated that smaller plots had higher species richness, mean *C*, and native perennial cover, and lower *Phragmites* cover than the large plots (Table 2 and Supplementary Figures S1–S4).

**Table 2:** Selection of predictor variables from each variable category for inclusion in variation partitioning.

Predictor variable	Plant community	Species richness	Mean C	*Phragmites*	Native perennials
**Scale**					
Spatial scale	#ns	X^∗∗^ –	X^∗∗∗^ –	X. +	X^∗^ –
**Management**					
Herbicide season	#ns	#ns	X. +	X^∗^ –	X. +
Herbicide type					
**Abiotic variables**					
PC1 (hydrology)	#ns	X^∗∗∗^ –	X^∗∗∗^ –	#ns	X^∗∗∗^ –
PC2 (nutrients)					
**Landscape variables**					
PC1 (hydrologic disturbance)	X^∗∗^	X^∗∗^ –		X. +	X^∗∗^ –
PC2 (developed disturbance)	X^∗^				
Proportion emergent marsh		X. +		X. –	
% cover surrounding natives	X^∗∗^	X^∗∗^ +	X^∗^ +		X^∗∗^ +

*Response variables were plant community composition, plot level species richness, mean conservation coefficient, *Phragmites* cover, and native perennial cover. Categorical factors were spatial scale (small plots: 0, large plots: 1), herbicide season (summer: 0, fall: 1), and herbicide type (imazapyr: 0, glyphosate: 1). ^X^ indicates that a variable was selected during the stepwise procedure. ^#^ indicates that the variable was manually selected (based on a minimal increase in AIC or adjusted *R*^2^) to ensure that at least one predictor from each category was present. Significance of terms in the final models is identified ^∗∗∗^*P* ≤ 0.001, ^∗∗^*P* ≤ 0.01, ^∗^*P* ≤ 0.05, *P* ≤ 0.1, ns > 0.1. For significant terms, ^*+*^ indicates a positive effect and ^-^ indicates a negative effect.*

### Variation Partitioning

Scale was a significant variable in explaining variation in each plant community outcome, but the amount of variation scale alone explained was minimal (<5%) (Table 3). For each univariate plant community outcome, the combined influence of scale and abiotic factors explained ∼14–38% of variation. Scale in combination with both landscape and abiotic variables explained 23% of variation for mean *C* and 11% of variation for native perennial cover (Table 3).

**Table 3:** Percentage variation explained by the unique contributions of scale (S), management (M), abiotic (A), and landscape (L) factors, and the variation explained by their intersections.

Explanatory variable set	Variance explained (%)				
	Plant community	Species richness	Mean *C*	*Phragmites* cover	Native perennials cover
S (Scale)	4.6.	0^∗∗^	0^∗∗∗^	3.8.	0^∗∗∗^
M (Management)	2.1	0	11.9	9.3.	3.2
A (Abiotic)	12.9^∗∗∗^	26.3^∗∗^	15.3^∗∗∗^	0	11.8^∗∗∗^
L (Landscape)	2.2^∗∗∗^	12.5^∗∗∗^	0^∗^	15.8.	6.8^∗∗∗^
SM	0	0	0	0	0
SA	0	38.2	22.3	13.9	30.8
SL	0.2	0	0	0	0
MA	0	0	0	0	0
ML	0	0.2	0.3	1.6	0
AL	2.2	0	0	0	6.4
SMA	0.1	0	0.8	0.23	0
SLA	0.2	0	23.4	0	11.0
MLA	0	0.2	0	0	0
SMLA	0	0	0	0	0
Residuals	77.6	42.9	32.2	71.1	34.0

*The significance of the testable model fractions (i.e., the unique contributions) was denoted by ^∗∗∗^*P* ≤ 0.001, ^∗∗^*P* ≤ 0.01, ^∗^*P* ≤ 0.05, *P*≤ 0.1.*

Management explained almost 10% of the variation for *Phragmites* cover, but was not a significant variable for any other response (Table 3). Fall herbicide treatments resulted in less *Phragmites* cover than summer treatments, but type of herbicide was not significant (Table 2 and Supplementary Table S6).

**FIGURE 5:**
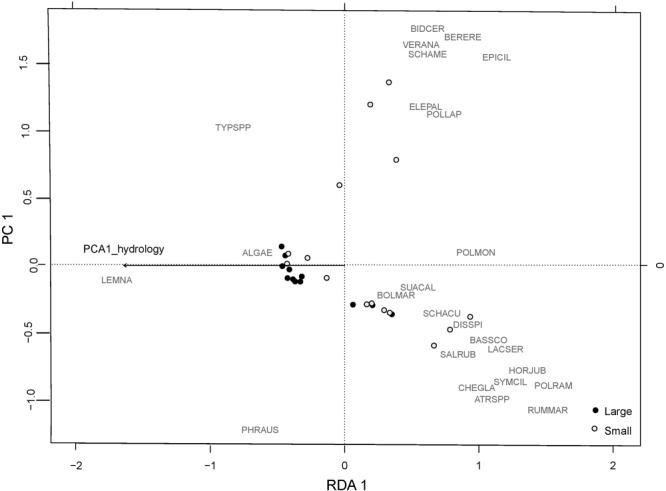
RDA plot of the plant community from 2015 with the PCA1 hydrology overlaid. Large plots are marked by black dots. Small plots are marked by open white dots. Species codes are: ALGAE: algae; ATRSPP: *Atriplex* spp.; BASSCO: *Bassia scoparia*; BERERE: *Berula erecta*; BIDCER: *Bidens cernua*; BOLMAR: *Bolboschoenus maritimus*; CHEGLA: *Chenopodium glaucum*; DISSPI: *Distichlis spicata*; ELEPAL: *Eleocharis palustris*; EPICIL: *Epilobium ciliatum*; HORJUB: *Hordeum jubatum*; LACSER: *Lactuca serriola*; LEMNA: *Lemna* spp.; POLLAP: *Polygonum lapathifolium*; POLMON: *Polypogon monspeliensis*; POLRAM: *Polygonum ramosissimum*; PHRAUS: *Phragmites australis*; RUMMAR: *Rumex maritimus*; SALRUB: *Salicornia rubra*; SCHACU: *Schoenoplectus acutus*; SCHAME: *Schoenoplectus americanus*; SUACAL: *Suaeda calceoliformis*; SYMCIL: *Symphyotrichum ciliatum*; TYPSPP: *Typha* spp.; VERANA: *Veronica anagallis-arvensis*.

Abiotic variables were consistently the most important unique variable explaining variation in plant community responses (Table 3). Deeper hydrology (higher values of abiotic PCA1) had a negative influence on native perennials, species richness, and mean *C* (Table 2 and Supplementary Table S6). The RDA biplot with the abiotic principal component overlaid showed patterns that reflected these results (Figure 5). Sites with low scores along the first axis were dominated by a few species with lower habitat value that could tolerate deep flooding including *Lemna* spp., *Typha* spp., and *Phragmites*. Sites with high scores along the first axis were associated with more diverse species assemblages, and organized by more drought tolerant species (i.e., *Salicornia rubra* and *Hordeum jubatum*) associated with high scores on axis 2, and more obligate wetland species (i.e., *Eleocharis palustris* and *Bidens cernua*) with low scores on axis 2.

**FIGURE 6:**
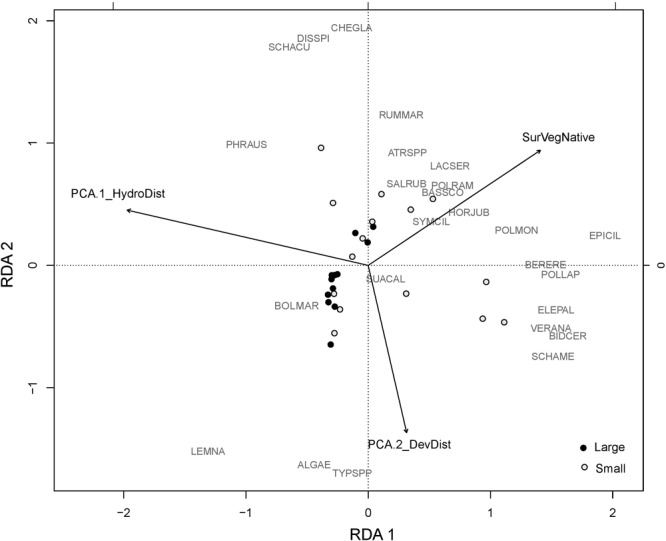
RDA of the plant community from 2015 with the significant landscape variables overlaid. Large plots are marked by black dots. Small plots are marked by open white dots. The RDA vectors are PCA.1_HydroDist: Landscape PCA 1 representing nearby hydrologic disturbances like canals and impoundments; PCA.2_DevDisturbance: Landscape PCA2 representing nearby disturbance from nearby human developments; SurVegNative: percent cover of native perennial species in the surrounding vegetation of each plot. For species codes, refer to Figure 5.

Landscape variables were significant for each plant community outcome, and uniquely explained ∼7–13% of variation in native perennials and species richness. Plots with high amounts of hydrologic disturbance in the landscape (high levels of landscape PCA1) had lower levels of species richness and perennial cover, while those that had high amounts of perennial natives in the surrounding landscape had high cover of native perennials and greater species richness (Table 2). Landscape variables also accounted for nearly half of the explained variation in *Phragmites* cover (∼16%) (Table 3). Plots with higher levels of hydrologic disturbance and with a smaller proportion of emergent marsh in the surrounding landscape had higher covers of *Phragmites* (Table 2). The RDA biplot with significant landscape variables overlaid reflected these results. Plots that were more dominated by *Phragmites* were associated with higher levels of hydrologic disturbance in the landscape (Figure 6). More species rich wetland plant communities were associated with greater amounts of native perennials in the landscape.

## Discussion

*Phragmites* is known to be one of the most problematic wetland invaders in North America (Galatowitsch et al., 1999) and is heavily managed (Martin and Blossey, 2013), yet little is known about how the size and environmental context of managed patches can influence *Phragmites* management outcomes (Hazelton et al., 2014; Quirion et al., 2018). In this study, we found that herbicide treatments led to more consistently successful outcomes in small patches compared with large ones. Over a 5-year management timeline, *Phragmites* reinvaded more slowly, and native perennial plants established at higher covers in the small patches. Like other studies that have evaluated patch scale influence on assembling communities (Ricketts, 2001; Holl and Crone, 2004), we found the scale of the treated patch alone accounted for very little of the variation in plant community responses. However, scale jointly with abiotic and/or landscape factors explained large amounts of variation in *Phragmites* and native plant responses following management. Specifically, large patches often had deeper and more prolonged flooding than small patches, which had a negative influence on the cover, conservation value, and richness of native plants. Large patches were also often in areas with more landscape-scale hydrologic disturbance, which promoted *Phragmites* reinvasion and had a negative influence on the cover and habitat quality of returning native species. Small patches were more often surrounded by a matrix of native perennial species, which had a positive influence on native plant recovery. For the most successful outcomes, managers should focus *Phragmites* treatments on patches with shallow flooding, less hydrologic disturbance, and more native species in the surrounding matrix.

### Phragmites Management Success at Two Spatial Scales

Invasive species reinvasion following management efforts is common, and has been attributed to variable environmental conditions, invader propagule pressure, native plant recruitment limitations, or ineffective treatments (Pearson et al., 2016). Patch scale can also have implications for invasive species reinvasion patterns. Mirroring our results, another recent study found *Phragmites* herbicide management most effective at smaller scales (Quirion et al., 2018), though they found the best *Phragmites* cover reduction in patches less than 1 m^2^, far smaller than our 1000 m^2^ small patches. *Phragmites* reinvasion, particularly following management of large patches (>5 acres), is a common observation among managers (Myers et al., 2009). We found that patch size alone accounted for a small amount of variation (<5%) in *Phragmites* cover, likely explained by the different modes of follow-up herbicide treatment at the different scales (machine herbicide spraying in large patches vs. more precise and less destructive backpack spraying in small patches). But most of the variation in *Phragmites* cover that we observed was from abiotic conditions and landscape influences, factors which often corresponded with patch scale.

*Phragmites* can withstand a wide range of salinity, hydrologic, and nutrient conditions (Eller et al., 2017), but treatments may not be equally effective across environmental gradients (Rohal, 2018). In this study, abiotic conditions in combination with scale accounted for a large amount of the variation in *Phragmites* cover following treatments. This result was largely explained by the differences in flooding observed across patch scales, with deeper water and larger organic horizons observed in large-scale plots (e.g., average water depth for large patches was 17.5 cm compared with 2.6 cm for small patches). *Phragmites* herbicide management is most effective when soil moisture is high, since drought-stressed *Phragmites* does not effectively translocate herbicides to the roots (Carlson et al., 2009; Rohal et al., 2017; Rohal, 2018). But water depth, particularly after management treatments cease, likely plays an important part in the competitive dynamics between *Phragmites* and other native species. For example, fewer plant competitors can withstand deeper water, thus increasing light resource availability and increasing ecosystem susceptibility to invasion (Davis et al., 2000). Established *Phragmites* is highly tolerant of inundation, while many native species cannot withstand these conditions (Brix et al., 1992; Eller et al., 2017). Flooding (>3.5 cm) is known to inhibit *Phragmites* seed germination (Baldwin et al., 2010; Kettenring et al., 2015), but also can limit the germination of many native wetland plants (Leck, 2003). Those patches that had deeper flooding likely saw an expansion of the remaining *Phragmites* through rhizomes due to clonal integration (Amsberry et al., 2000), with limited recruitment of *Phragmites* by seed, but also limited recruitment of native species. Lower native plant recruitment likely also limited the biotic resistance to *Phragmites* expansion (Byun et al., 2015), which in turn may have contributed to the higher amounts of *Phragmites* observed in the large flooded patches. Very deep flooding (>0.5 m) can restrict *Phragmites* growth (Hudon et al., 2005), but the depth of flooding we measured (the deepest plots rarely exceeded 30 cm) did not limit *Phragmites* expansion after herbicide treatments ceased.

The environmental factors and processes that lead to the creation of large, expansive *Phragmites* patches are explored in the literature, but are poorly understood (Lathrop et al., 2003; Kettenring et al., 2016). *Phragmites* patch expansion rates and patch dynamics are highly variable across its range (reviewed in Kettenring et al., 2016), indicating that the creation of large patches is not just a function of time since invasion, but may also be influenced by other variables, such as environmental factors, propagule pressure, and disturbance histories. It is often suggested that site hydrology may play an important role (Warren et al., 2001; Kettenring et al., 2016). Given the stark differences in hydrology that we observed in the small and large patches, we hypothesize that it may be altered hydrologic patterns [specifically prolonged flooding (<0.5 m) through the growing season] that most enable the rapid patch expansion and the creation of large patches. This study highlights that these conditions, in turn, can limit restoration outcomes.

*Phragmites* is a high nutrient specialist (Mozdzer et al., 2010); with plentiful nutrients it can produce more above-ground biomass (Minchinton and Bertness, 2003), greater numbers of florets and inflorescences (Kettenring et al., 2011), and explosive seedling growth (Kettenring and Whigham, 2018). Contrary to our expectations, soil nutrients did not explain any of the variation in *Phragmites* reinvasion following management. Our one-time measurements of soil nutrients likely did not capture temporal and spatial variation in nutrient dynamics or all the different forms of nitrogen important to *Phragmites* growth (Mozdzer et al., 2010), which may have been more important in explaining vegetation dynamics. High salinity can also constrain *Phragmites* growth (Burdick et al., 2001), and may have an influence on treatment effectiveness when elevated, but the salinities observed in our treatment plots were well within its tolerance limits (Eller et al., 2017).

Landscape level disturbances are widely considered factors that can promote plant invasions (Menuz and Kettenring, 2013), particularly in wetlands that are landscape sinks (Zedler and Kercher, 2004). Our findings indicate that some landscape-scale disturbances, such as hydrologic disturbances from roads and canals associated with man-made impoundments can also promote the reinvasion of *Phragmites* following management. Landscape factors associated with disturbance to a wetland’s natural hydrology, including shoreline alterations, dredging, and diking, are often implicated in promoting invasions (Galatowitsch et al., 1999), and have been linked to *Phragmites* invasions (Chambers et al., 1999, 2003). Restrictions to natural hydrology may create lower salinities than unaltered wetlands, which can promote *Phragmites* presence (Burdick et al., 2001). Impoundments and water alterations also likely contributed to the deeper and longer duration flooding that can contribute to *Phragmites* competitive dominance (Brix et al., 1992; Galatowitsch et al., 1999; Kercher and Zedler, 2004). Despite the possible negative implications, impoundments have enormous benefits for buffering GSL wetlands from drought, since water availability is limited by widespread upstream urban and agricultural uses (Downard and Endter-Wada, 2013). Water control associated with such impoundments can often assist *Phragmites* management efforts by allowing for increased equipment access (Rohal et al., 2017). In addition, water control may allow for the manipulation of a hydrologic regime that could favor native species over *Phragmites*, though this has been unexplored empirically.

### Native Plant Recovery Following Phragmites Management

Despite minimal quantitative evidence of plant community outcomes following *Phragmites* management (Hazelton et al., 2014), *Phragmites* managers often assume that conditions will be adequate for native plant recruitment following management (Rohal et al., 2018). However, we saw variable levels of native plant return following management efforts, with consistently greater covers of native perennial plants and higher species richness in small patches than large (Supplementary Table S5). The size of the patch alone accounted for very little of this variation in plant communities. While scale-based ecological processes like differing spatial heterogeneity and edge to area ratios were likely still at play (Englund and Cooper, 2003; Holl and Crone, 2004), they were less important relative to abiotic and landscape factors at the temporal scale of this study. Spatial scale in combination with abiotic conditions (predominantly hydrology) explained the most variation for each measured plant community response.

Hydrology, specifically flooding depth, duration, and frequency, is known to be a factor of overriding influence in determining plant community assembly in wetlands (Reddy and DeLaune, 2008). Thus, our finding that hydrologic variables were the leading driver of plant community outcomes is not surprising and confirms similar findings in other wetlands (e.g., Matthews et al., 2009; Tousignant et al., 2010; Tsai et al., 2012). Like others studying arid wetlands (Tsai et al., 2012), we found that greater water depth and increased organic horizons (associated with prolonged flooding) negatively influenced species richness and native perennial cover. Shallowly flooded or mudflat conditions that promote a more diverse suite of wetland plant germination syndromes (Smith and Kadlec, 1983) were more common in the small patches, where we recorded greater numbers of species and covers of native perennials. Deep and prolonged flooding in the large patches also negatively influenced the conservation value of the returning species. *Typha*, a species with low conservation value and considered a wetland invader in many ecosystems, was the most dominant emergent plant beyond *Phragmites* that returned to large, more deeply flooded patches. Like *Phragmites, Typha* is more tolerant of deeply flooded conditions than many native species (Kercher and Zedler, 2004). Hydrologic shifts that lead to deep and prolonged flooding often favor invasive species that are more broadly tolerant of these altered conditions (Zedler and Kercher, 2004).

Still, others have found that dispersal and seed availability are more limiting than abiotic conditions in wetland plant assembly (Zobel et al., 2000; Kettenring and Galatowitsch, 2011a,b), suggesting seed availability might also be limiting the plant community responses we observed. Even in more deeply flooded sites, the heterogeneity we observed in water depth was substantial, which indicates that there were enough germination opportunities to expect greater richness and covers of perennial wetland species than what we observed. For example, three bulrush species (*Schoenoplectus americanus, S. acutus*, and *B. maritimus*) that are the dominant perennial species in GSL (Downard et al., 2017) all have superior germination in 5–10 cm of water (Clevering, 1995), conditions that were met in all but the most deeply flooded sites. If these species were readily available in the seed bank, we would expect to see them take advantage of microsites, yet we observed minimal recruitment, possibly because the densities of invaded seed banks rarely match uninvaded areas (Gioria and Pyšek, 2015). While seed banks under *Phragmites* stands are diverse in tidal systems (Baldwin et al., 2010; Hazelton et al., 2018), this may not be the case in inland systems, particularly where hydrologic connectivity between wetlands has been disrupted.

Dispersal and connectivity between a patch and native plants in the surrounding area may mediate the degree to which assembling plant communities reflect underlying environmental gradients (Alexander et al., 2012). We found that disturbance in the landscape associated with impoundment infrastructure, including roads and canals, was associated with less native perennial cover and species richness, a result that might be partially explained by the negative influence of hydrologic disturbance on propagule dispersal. Dams, ditching, and diking are known to disrupt seed dispersal, particularly for water transported seed (Nilsson et al., 2010) like those of many high-quality wetland perennials in GSL (Kadlec and Smith, 1984). Disturbance to the natural hydrologic regime associated with impoundment infrastructure might also reduce establishment opportunities by restricting drawdown conditions (Van Geest et al., 2005).

While constricted dispersal routes can negatively influence wetland plant assembly, limitations in the abundance and distribution of propagules in the surrounding area can also constrain plant community assembly in wetlands (Houlahan et al., 2006). The absence of native species surrounding large patches may have contributed to the poor native plant recruitment we observed in such areas. We found a positive relationship between the cover of native perennials in the surrounding matrix and the cover and richness of native species assembling in treated patches, which indicates that the increased native propagule pressure associated with such conditions promotes more robust native recruitment following *Phragmites* management. These results are in line with many other studies that found positive associations between proximity to native populations and the richness and cover of desirable plant communities following invasive plant removal (e.g., Erskine Ogden and Rejmánek, 2005; Matthews et al., 2017) and other restoration actions (e.g., Holl and Crone, 2004; Helsen et al., 2013). We found a positive relationship between native perennials in patch surroundings and the conservation value of returning plant communities. An intact native matrix may be evidence of less disturbance (Reid et al., 2009), conditions which can promote the assembly of higher quality native plant species (Cohen et al., 2004).

### Management Implications

While managers seek to refine management practices to achieve restoration goals, it is often factors outside of management decision-making, like site, landscape, or historical contingencies that drive plant community outcomes (Brudvig, 2011). In this study, management choices about herbicide type and timing were less important than landscape and abiotic factors for explaining differences in *Phragmites* cover and native plant recovery following treatments. Nevertheless, while herbicide type was insignificant, fall herbicide timing did result in less *Phragmites* cover and led to more consistent results across scales than summer treatments. Fall treatments were superior for *Phragmites* removal likely because herbicide is more effectively translocated to rhizomes in the fall as *Phragmites* prepares for senescence (Tu et al., 2001). While fall treatments released more opportunities for native establishment by more effectively removing *Phragmites*, herbicide timing did not explain much variation in plant community responses. Although we did not see a strong signal for management decisions on plant community outcomes in this study, we caution that management decisions may still have important consequences in other contexts. We chose management methods that we expected would be effective, following consultations with experienced land managers and a review of previous research (Rohal et al., 2018). Had we included less appropriate management actions, such as manual removal without herbicide, we may have seen greater variability in plant community outcomes, and a stronger signal for management.

Multiple ecological models have supported recommendations for managers to target small, outlier patches of invasive species, which most efficiently reduces invader spread and is more cost effective (Moody and Mack, 1988; Higgins et al., 2000; Taylor and Hastings, 2004). One *Phragmites*-specific model offered evidence that prioritizing small patches of *Phragmites* is more effective for reducing spread (Alminagorta Cabezas, 2015), explained by *Phragmites*’ ability to rapidly expand vegetatively (Kettenring et al., 2016), and the larger edge to area ratios of small patches. Regardless, large patches of *Phragmites* are often targeted, though this may not be the best method to reduce *Phragmites* cover at the landscape scale. For example, one 6-year *Phragmites* monitoring effort showed that the reduction of *Phragmites* cover from the herbicide treatment of large patches (>5 acres) was offset by rapid expansion of untreated small patches (Myers et al., 2009). In addition, our study, like Quirion et al. (2018), provides evidence that large patches of *Phragmites* might also be more challenging to manage effectively, and may result in lower recruitment of native plants. Our research provides further context, suggesting that it is not the scale of the patch, *per se*, that controls outcomes, but the abiotic and landscape factors that correspond with patch scale that drive plant community results. Thus, to maximize plant community results, managers should target small *Phragmites* patches in less disturbed areas, with substantial established native communities in the matrix (Long et al., 2017b). With limited resources, choosing less degraded sites (Reid et al., 2009; Prior et al., 2017) with a matrix dominated by desirable native species (Matthews et al., 2017) allows managers to maximize the success of their efforts (Holl and Aide, 2011).

Often the management of large patches of invasive species is prioritized because of political reasons (Palmer, 2009), feasibility (Larson et al., 2011), and site-specific concerns (McGeoch et al., 2016). Should practitioners choose to manage large patches of *Phragmites*, our results suggest they should then plan for the additional constraints that are likely associated with such patches. Particularly for large patches that are isolated from intact native species, or subject to hydrologic disturbance that might limit native species dispersal, managers should consider including active revegetation in management plans, which can help overcome propagule limitations (Erskine Ogden and Rejmánek, 2005; Morzaria-Luna and Zedler, 2007) and promote more rapid native recruitment, increasing biotic resistance to *Phragmites* reinvasion (Byun et al., 2015). If hydrologic control is an option, our research suggests promoting a more natural hydrologic pattern instead of moderately deep and prolonged flooding, which can promote more native species germination. However, managers should be cautious that these conditions might also promote *Phragmites* germination (Kettenring et al., 2015).

Invasive plant experiments are often conducted at small (<1 m^2^) scales which can limit their applicability to real-world restorations (Kettenring and Adams, 2011). Like Erskine Ogden and Rejmánek (2005), we found that the results we observed at a smaller scale did not translate to large-scale efforts, particularly regarding native plant recovery after invader removal. These results highlight the importance of having an experimental arena that reflect the common scales of management (Englund and Cooper, 2003). And it supports the growing call (Brudvig et al., 2017) to conduct restoration research across many sites with diverse abiotic, historic, and landscape contexts to better understand the contingencies of restoration outcomes. Partnerships between researchers and managers can enable investigation at the necessary spatial and temporal scales to elucidate such constraints (Zedler, 2000; Rohal et al., 2018).

## Author Contributions

All authors contributed substantially to this work. CR, CC, and KK contributed to the experimental design, data acquisition, and data interpretation. CR analyzed data and drafted the manuscript. KK contributed critical revisions and oversight of this work.

## Conflict of Interest Statement

The authors declare that the research was conducted in the absence of any commercial or financial relationships that could be construed as a potential conflict of interest.
