# Supplementary material for: Abiotic and Landscape Factors Constrain Restoration Outcomes Across Spatial Scales of a Widespread Invasive Plant
Source: Front Plant Sci. 2019 Apr 18;10:481. doi: 10.3389/fpls.2019.00481 (PMC6499188; doi:10.3389/fpls.2019.00481)
Supplement: Supplementary file 1 [file Table_1.docx]

Supplementary Material

**Abiotic and Landscape Factors Constrain Restoration Outcomes Across Spatial Scales of a Widespread Invasive Plant**

Christine B. Rohal*, Chad Cranney, Karin M. Kettenring

*Correspondence: Corresponding author: [christine.rohal@gmail.com](mailto:christine.rohal@gmail.com)

**Supplementary Tables and Figures**

**Supplementary Table S1**. Pearson’s correlations between predictor variables.

|  | **Scale** | HSeas | HTyp | PCA1A | PCA2A | PCA1L | PCA2L | SurVegNat |
| --- | --- | --- | --- | --- | --- | --- | --- | --- |
| **Management** |  |  |  |  |  |  |  |  |
| H Season | -0.01 |  |  |  |  |  |  |  |
| H Type | 0.00 | 0.50 |  |  |  |  |  |  |
| **Abiotic** |  |  |  |  |  |  |  |  |
| PCA1_A | -0.81 | 0.00 | 0.01 |  |  |  |  |  |
| PCA2_A | 0.26 | 0.08 | -0.09 | 0.00 |  |  |  |  |
| **Landscape** |  |  |  |  |  |  |  |  |
| PCA1_L | -0.32 | -0.03 | 0.05 | 0.43 | 0.06 |  |  |  |
| PCA2_L | 0.16 | 0.09 | 0.14 | -0.10 | 0.02 | 0.00 |  |  |
| SurVegNative | 0.66 | -0.06 | 0.12 | -0.71 | -0.20 | -0.29 | 0.29 |  |
| Emergent Marsh | -0.32 | 0.08 | 0.04 | 0.31 | -0.02 | -0.11 | -0.31 | -0.25 |

**Supplementary Table S2.** *Phragmites* cover effect sizes. Significance of terms are identified *** ≤0.001, ** ≤0.01, * ≤0.05.

|  |  | **Small scale** | | | **Large scale** | | | | | |  | | |  |
| --- | --- | --- | --- | --- | --- | --- | --- | --- | --- | --- | --- | --- | --- | --- |
|  |  |  | **95% CI** | |  |  | | **95% CI** | | | |  | | |
| **Treatment** | **Year** | **Effect size** | **Lower** | **Upper** | **Effect size** | | **Lower** | | **Upper** | ***Q_M_*** | | |  |  |
| Fall glyphosate | 2013 | -1.53*** | -2.08 | -0.98 | -1.95*** | | -2.59 | | -1.31 | 0.95 | | |  |  |
|  | 2014 | -0.75** | -1.21 | -0.29 | -1.15* | | -2.08 | | -0.23 | 0.52 | | |  |  |
|  | 2015 | -1.29*** | -1.60 | -0.97 | -1.27* | | -2.22 | | -0.32 | 0.17 | | |  |  |
|  | 2016 | -0.85* | -1.54 | -0.15 | -0.73*** | | -1.07 | | -0.39 | 0.03 | | |  |  |
| Summer glyphosate | 2013 | -0.90* | -1.68 | -0.12 | -1.94*** | | -2.68 | | -1.19 | 3.63 | | |  |  |
|  | 2014 | -0.84* | -1.48 | -0.20 | -1.33*** | | -1.70 | | -0.96 | 2.28 | | |  |  |
|  | 2015 | -0.94* | -1.68 | -0.20 | 0.14 | | -0.47 | | 0.74 | 4.85* | | |  |  |
|  | 2016 | -0.41** | -0.71 | -0.11 | -0.07 | | -0.19 | | 0.06 | 4.57* | | |  |  |
| Summer imazapyr | 2013 | -2.18** | -3.59 | -0.78 | -2.34*** | | -3.40 | | -1.29 | 0.05 | | |  |  |
|  | 2014 | -1.60*** | -2.44 | -0.77 | -2.00* | | -3.94 | | -0.05 | 0.03 | | |  |  |
|  | 2015 | -1.91 | -4.00 | 0.17 | -0.18 | | -1.12 | | 0.75 | 2.21 | | |  |  |
|  | 2016 | -1.23 | -2.52 | 0.06 | -0.40 | | -0.98 | | 0.19 | 1.23 | | |  |  |

**Supplementary Table S3.** Native emergent perennial cover effect sizes. Significance of terms are identified *** ≤0.001, ** ≤0.01, * ≤0.05.

|  |  | **Small scale** | | | **Large scale** | | | |  | |
| --- | --- | --- | --- | --- | --- | --- | --- | --- | --- | --- |
|  |  |  | **95% CI** | |  | **95% CI** | |  | |  |
| **Treatment** | **Year** | **Effect size** | **Lower** | **Upper** | **Effect size** | **Lower** | **Upper** | ***Q_M_*** | |  |
| Fall glyphosate | 2013 | 2.23 | -0.08 | 4.43 | 0.62 | -0.67 | 1.92 | 1.34 | |  |
|  | 2014 | 3.78** | 1.20 | 6.46 | 4.29 | -0.38 | 8.96 | 0.04 | |  |
|  | 2015 | 6.97*** | 3.64 | 10.30 | 3.87 | -1.07 | 8.82 | 1.00 | |  |
|  | 2016 | 4.67*** | 2.13 | 7.20 | 4.37 | -0.86 | 9.60 | 0.01 | |  |
| Summer glyphosate | 2013 | 1.54 | -0.65 | 3.74 | 1.34 | -0.46 | 3.14 | 0.02 | |  |
|  | 2014 | 4.33 | -0.58 | 9.24 | 2.29 | -2.65 | 7.24 | 0.33 | |  |
|  | 2015 | 7.02** | 2.58 | 11.45 | 1.16 | -2.75 | 5.07 | 3.79* | |  |
|  | 2016 | 3.59 | -0.96 | 8.13 | 1.01 | -3.28 | 5.29 | 0.66 | |  |
| Summer imazapyr | 2013 | 2.73 | -0.71 | 6.18 | 2.29 | -1.32 | 5.90 | 0.03 | |  |
|  | 2014 | 5.14 | -0.46 | 10.75 | 1.97 | -3.82 | 7.75 | 0.60 | |  |
|  | 2015 | 6.94** | 2.17 | 11.70 | 2.92 | -2.79 | 8.62 | 1.11 | |  |
|  | 2016 | 5.11** | 1.60 | 8.61 | 3.64 | -1.67 | 8.96 | 0.19 | |  |

**Supplementary Table S4.** Species richness effect sizes. Significance of terms are identified *** ≤0.001, ** ≤0.01, * ≤0.05. Species richness was calculated at the plot level, which reduced sample size, making the Qm statistic unable to be calculated for this metric.

|  |  | **Small Scale** | | | **Large Scale** | | | | | | | |  | | |  |
| --- | --- | --- | --- | --- | --- | --- | --- | --- | --- | --- | --- | --- | --- | --- | --- | --- |
|  |  |  | **95% CI** | |  |  | | **95% CI** | | | | | |  | | |
| **Treatment** | **Year** | **Effect size** | **Lower** | **Upper** | **Effect size** | | **Lower** | | **Upper** | | |  | | |  |  |
| Fall Glyphosate, Winter Mow | 2013 | 0.94** | 0.38 | 1.51 | 0.62 | | -0.18 | | | 1.42 |  | | | |  |  |
|  | 2014 | 1.07* | 0.21 | 1.94 | 1.05*** | | 0.53 | | | 1.57 |  | | | |  |  |
|  | 2015 | 2.04*** | 1.49 | 2.58 | 1.06** | | 0.34 | | | 1.77 |  | | | |  |  |
|  | 2016 | 1.63*** | 0.94 | 2.32 | 1.09** | | 0.41 | | | 1.79 |  | | | |  |  |
| Summer Glyphosate, Winter Mow | 2013 | 0.97** | 0.28 | 1.66 | 0.76* | | 0.03 | | | 1.50 |  | | | |  |  |
|  | 2014 | 1.21* | 0.28 | 2.14 | 0.94** | | 0.34 | | | 1.55 |  | | | |  |  |
|  | 2015 | 2.06*** | 1.46 | 2.65 | 0.97* | | 0.16 | | | 1.77 |  | | | |  |  |
|  | 2016 | 1.42*** | 0.64 | 2.20 | 0.77 | | -0.12 | | | 1.66 |  | | | |  |  |
| Summer Imazapyr, Winter Mow | 2013 | 0.83** | 0.30 | 1.36 | 0.54 | | -0.23 | | | 1.30 |  | | | |  |  |
|  | 2014 | 1.07* | 0.24 | 1.91 | 1.15*** | | 0.55 | | | 1.74 |  | | | |  |  |
|  | 2015 | 1.95*** | 1.44 | 2.45 | 0.69 | | -0.14 | | | 1.52 |  | | | |  |  |
|  | 2016 | 1.53*** | 0.92 | 2.14 | 0.69 | | -0.14 | | | 1.53 |  | | | |  |  |

**Supplementary Table S5.** Means plus standard errors of vegetation metrics in small and large *Phragmites* treatment plots. Vegetation metrics were percent cover *Phragmites*, percent cover native perennials, species richness, and mean C.

|  | **Control** | | **Fall**  **glyphosate** | | **Summer glyphosate** | | **Summer**  **imazapyr** | |
| --- | --- | --- | --- | --- | --- | --- | --- | --- |
|  | **Small** | **Large** | **Small** | **Large** | **Small** | **Large** | **Small** | **Large** |
| % cover *Phragmites* | 81.2±4.3 | 66.2±18.3 | 18.6±4.4 | 15.9±4.9 | 33.9±13.5 | 62.3±9.4 | 29.9±10.7 | 52.2±17.4 |
| % cover native perennials | 0.8±0.8 | 1.1±1.1 | 21.6±12.3 | 2.5±2.2 | 9.9±5.3 | 0.34±0.2 | 19.1±15.9 | 1.7±1.5 |
| Species richness | 1.2±0.2 | 2±0.6 | 9.2±2.1 | 5.8±1.1 | 9.4±2.4 | 5.3±1.4 | 8.4±1.6 | 4±1.2 |
| Mean C | 0.8±0.8 | 1.4±0.7 | 4.3±0.2 | 3.6±0.4 | 3.5±0.3 | 2.5±0.6 | 3.4±0.4 | 2.7±0.4 |

**Supplementary Table S6.** Statistical significance of predictor variables selected from each variable set (scale, management, abiotic, and landscape) for plant community, species richness, mean C, *Phragmites* cover, and native perennials cover. Open spots indicate that the variable was not selected to be included in the final model.

| **Predictor Variable** | **Plant community** | **Species richness** | **Mean C** | ***Phragmites*** | **Native perennials** |
| --- | --- | --- | --- | --- | --- |
| **Scale** |  |  |  |  |  |
| Spatial scale | #ns | F_1,25_=19.2  P=0.004 | F_1,25_=20.6  P<0.001 | F_1,25_=2.6  P=0.12 | F_1,25_=17.3  P<0.001 |
| **Management** |  |  |  |  |  |
| Herbicide season | #ns | F_1,25_=0.4  P=0.53 | F_1,25_=3.6  P=0.07 | F_1,25_=6.9  P=0.01 | F_1,25_=1.2  P=0.28 |
| Herbicide type |  |  |  |  |  |
| **Abiotic Variables** |  |  |  |  |  |
| PC1 (hydrology) | #ns | F_1,25_=24.0  P<0.001 | F_1,25_=34.9  P<0.001 | F_1,25_=1.5  P=0.23 | F_1,25_=36.4  P=<0.001 |
| PC2 (nutrients) |  |  |  |  |  |
| **Landscape Variables** |  |  |  |  |  |
| PC1 (hydrologic disturbance) | F=3.56  P=0.002 | F_1,23_=12.2  P=0.002 |  | F_1,24_=3.3  P=0.08 | F_1,24_= 11.1  P=0.002 |
| PC2 (developed disturbance) | F=2.39  P=0.018 |  |  |  |  |
| Proportion emergent marsh |  | F_1,23_=2.7  P=0.11 |  | F_1,24_=2.7  P=0.11 |  |
| % cover surrounding natives | F=3.34  P=0.002 | F_1,23_=10.7  P=0.003 | F_1,25_=6.7  P=0.02 |  | F_1,24_=13.7  P=0.001 |

| % cover native perennials (logit)  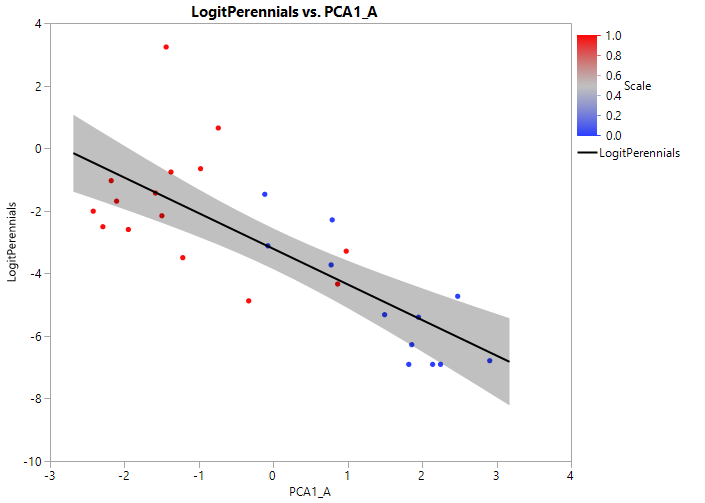 |
| --- |
| PCA1_Abiotic |
| % cover native perennials (logit)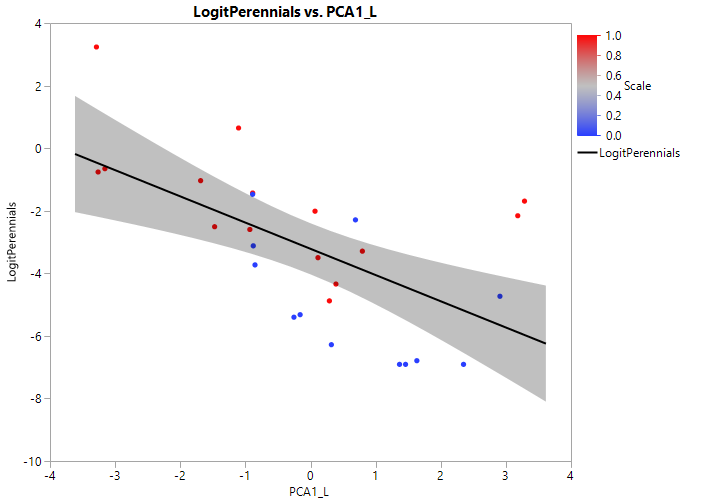 |
| PCA1_Landscape |
| % cover native perennials (logit)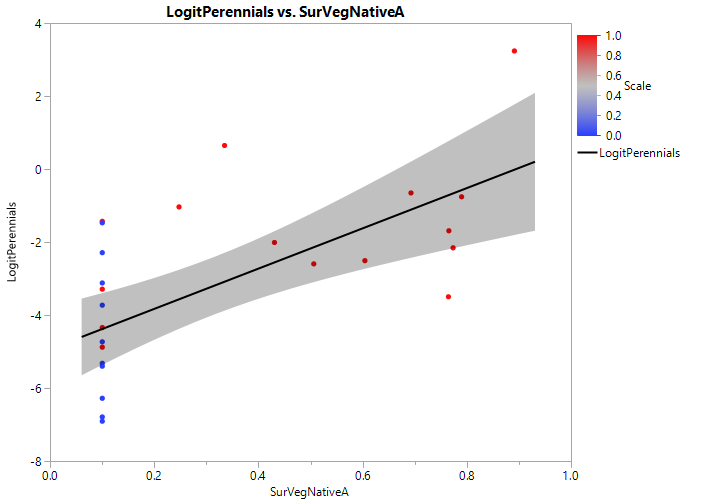 |
| % cover surrounding native perennials (arcsin sqrt) |

**Supplementary Figure S1**. Logit transformed native perennials plotted against abiotic and landscape variables used in variation partitioning. Abiotic and landscape variables are Abiotic PCA1, Landscape PCA1, and cover of surrounding native vegetation. Red points represent data from small plots, blue dots represent data from large plots.

| % cover *Phragmites* (logit)  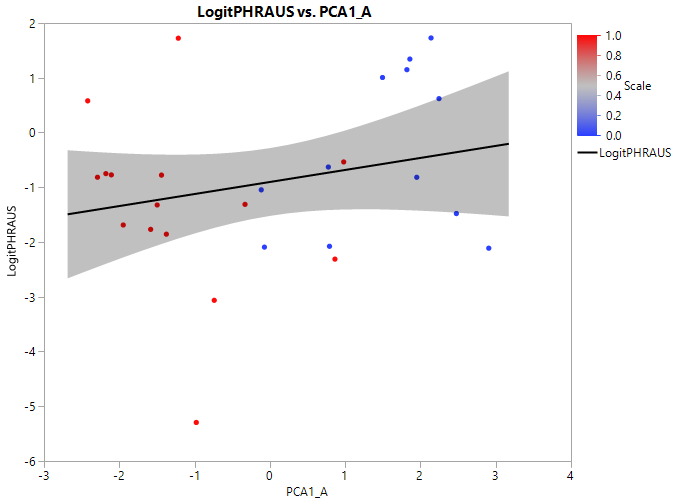 |
| --- |
| PCA1_Abiotic |
| % cover *Phragmites* (logit) 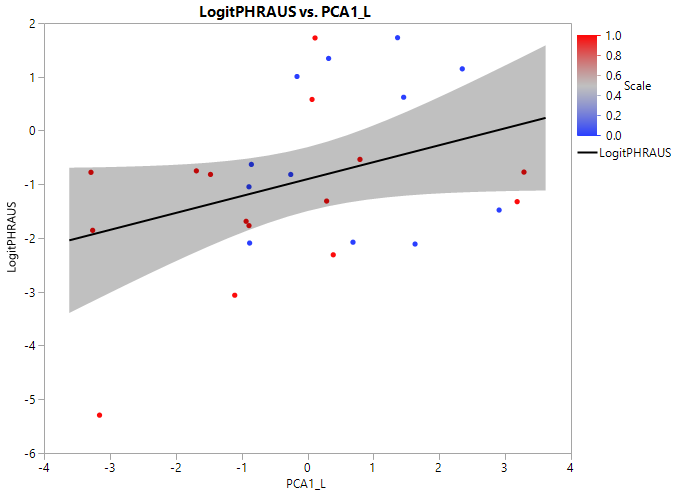 |
| PCA1_Landscape |
| % cover *Phragmites* (logit) 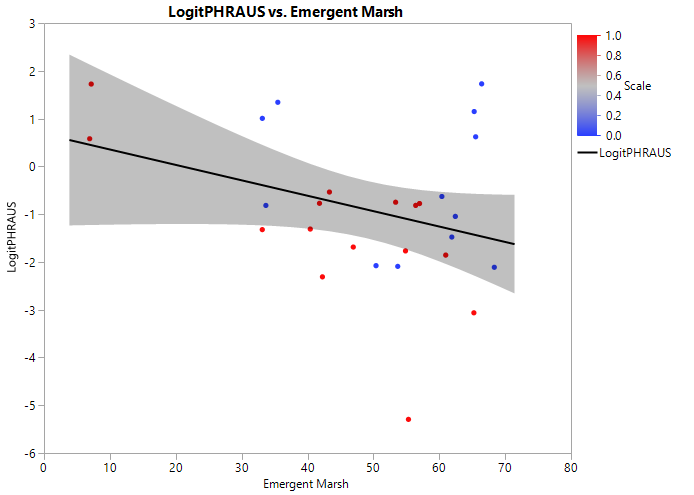 |
| Proportion emergent marsh in landscape |

**Supplementary Figure S2.** Logit transformed *Phragmites* cover plotted against abiotic and landscape variables used in variation partitioning. Variables included Abiotic PCA1, Landscape PCA1, and proportion emergent marsh in landscape. Red points represent data from small plots, blue dots represent data from large plots.

| Species richness  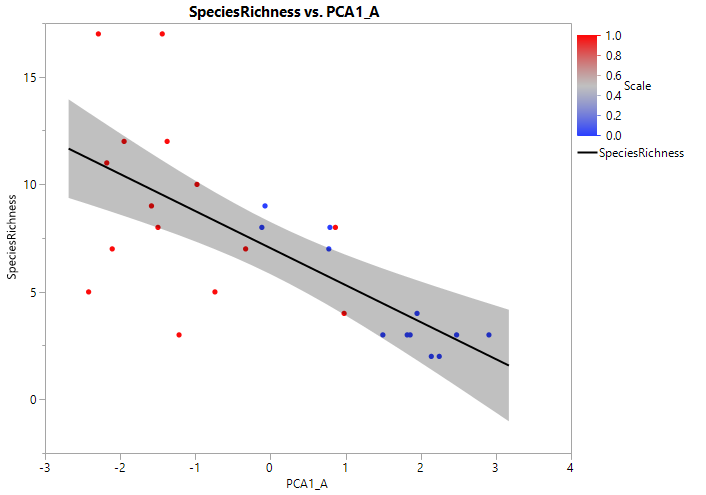 | Species richness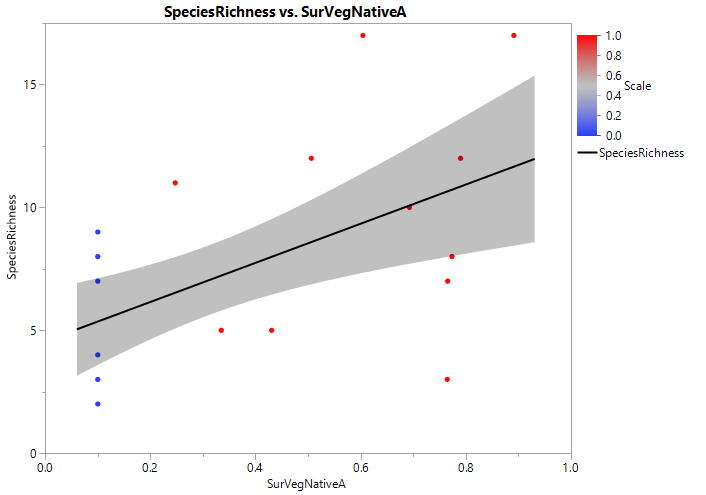 |
| --- | --- |
| PCA1_Abiotic | Surrounding veg native perennials |
| Species richness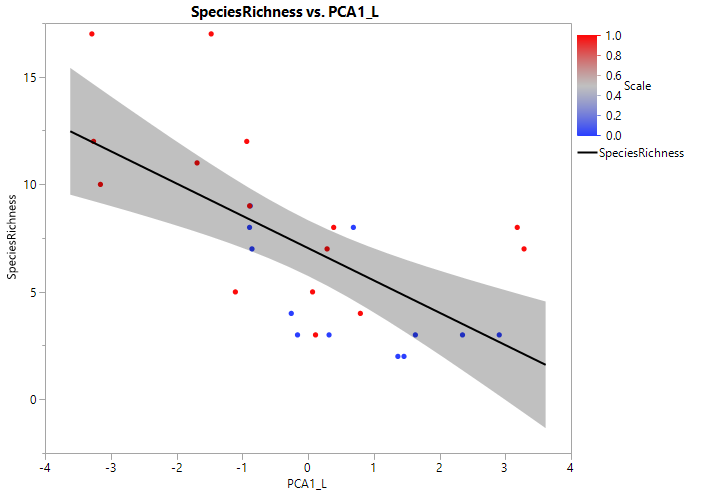 | Species richness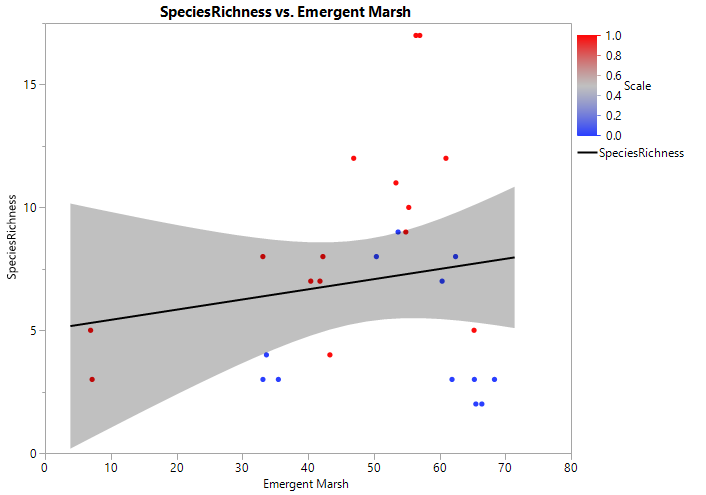 |
| PCA1_Landscape | Proportion emergent marsh in landscape |

**Supplementary Figure S3.** Species richness plotted against abiotic and landscape variables used in variation partitioning. Variables included Abiotic PCA1, Landscape PCA1, surrounding vegetation native perennials, and proportion emergent marsh in landscape. Red points represent data from small plots, blue dots represent data from large plots.

| Mean C  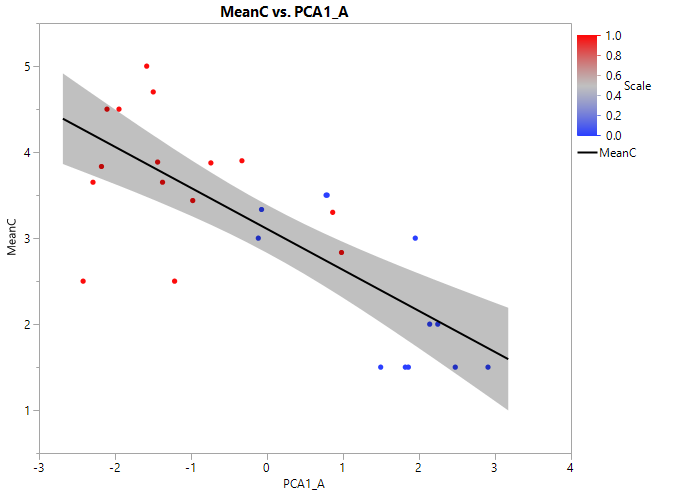 |
| --- |
| PCA1_Abiotic |
| Mean C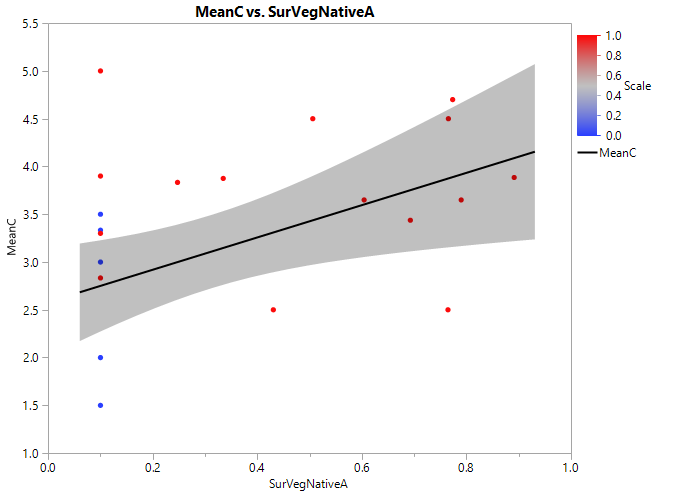 |
| Surrounding veg native perennials |

**Supplementary Figure S4.** Mean C plotted against abiotic and landscape variables used in variation partitioning. Variables included Abiotic PCA1, surrounding vegetation native perennials. Red points represent data from small plots, blue dots represent data from large plots.
